# The Current State of Knowledge on *Ribes* spp. (Currant) Plants

**DOI:** 10.3390/plants14203196

**Published:** 2025-10-17

**Authors:** Elnura Y. Izteleuova, Gulsim T. Zhumashova, Tolkyn S. Bekezhanova, Zoya B. Allambergenova, Karlygash A. Zhaparkulova, Aigerim A. Karaubayeva, Aigul K. Kaldybayeva, Zuriyadda B. Sakipova, Liliya N. Ibragimova, Izabela Korona-Glowniak

**Affiliations:** 1 Department of Engineering Disciplines and Good Practices, School of Pharmacy, Kazakh National Medical University named after S.D. Asfendiyarov, Tole Bi Street 94, Almaty 050000, Kazakhstan; bekezhanova.t@kaznmu.kz (T.S.B.); zoyaallambergen@mail.ru (Z.B.A.); karaubayeva.a@kaznmu.kz (A.A.K.); sakipova.z@kaznmu.kz (Z.B.S.); 2 Department of Pharmaceutical and Toxicological Chemistry, School of Pharmacy, Kazakh National Medical University named after S.D. Asfendiyarov, Tole Bi Street 94, Almaty 050000, Kazakhstan; g.zhumashova@mail.ru (G.T.Z.); aigul_240873@mail.ru (A.K.K.); 3 Department of Biotechnology and General Chemical Technology, School of Pharmacy, Kazakh National Medical University named after S.D. Asfendiyarov, Tole Bi Street 94, Almaty 050000, Kazakhstan; zhaparkulova.k@kaznmu.kz; 4 The Center for Pharmacy and Pharmacology, Science and Technology Park, Kazakh National Medical University named after S.D. Asfendiyarov, Tole Bi Street 94, Almaty 050000, Kazakhstan; ibragimova.l@kaznmu.kz; 5 Department of Pharmaceutical Microbiology, Medical University of Lublin, ul. Chodzki 1, 20-093 Lublin, Poland

**Keywords:** genus *Ribes* L., *Grossulariaceae*, phytochemical study, biologically active substances, pharmacological action

## Abstract

This review systematically compiles and evaluates current research on the ethnopharmacology, phytochemistry, pharmacological effects, and the clinical potential of plants of the genus *Ribes* L. *Ribes* species have long been used in traditional medicine for cardiovascular diseases, hepatitis, gastrointestinal ailments, hyperlipidemia, and detoxification, owing to their rich content of phenolic glycosides, flavonoids, proanthocyanidins, and polysaccharides. We analyze data from in vitro, in vivo, and clinical studies, focusing on antioxidant, anti-inflammatory, antibacterial, antiviral, antitumor, and vision-protective properties of extracts and isolated compounds. Particular attention is given to *R. nigrum*, a species recognized in French and British pharmacopeias for its antioxidant, anti-inflammatory, antimicrobial, and immunomodulatory properties. Our synthesis reveals significant gaps: many *Ribes* spp. remain poorly characterized chemically; toxicology and pharmacokinetics are seldom studied; clinical trials are limited in number and rigor. We propose that future research should prioritize the comprehensive metabolomic and chemoprofiling of understudied *Ribes* species; standardized safety and dosage studies; the elucidation of absorption, distribution, metabolism, and excretion (ADME) of key bioactives; and well-designed randomized clinical trials to validate traditional uses and establish therapeutic efficacy. Our review demonstrates that *Ribes* spp. are promising sources for novel phytopharmaceutical agents and functional foods, but that translational and regulatory research is essential to move from traditional claims toward evidence-based medical applications.

## 1. Introduction

Phytotherapy is a branch of medicine that utilizes plants for disease treatment and health promotion. In Western medicine, it is often known as herbalism. Traditional phytotherapy maintains the natural composition and integrity of the source plant, using either the whole plant or a specific portion of its minimally processed components for medicinal purposes. The World Health Organization (WHO) and other global health agencies have recently launched initiatives to promote traditional and complementary/alternative medicine for enhancing public health and well-being. A key priority for the WHO is ensuring the safe use of these therapies. Currants (*Ribes* spp.) have been found to offer beneficial effects in the dietary management of various diseases, including hypertension, osteoporosis, inflammation, cancer, and cardiovascular disease [1]. The genus *Ribes* L. belongs to the family of dicotyledonous flowering plants Gooseberry (*Grossulariaceae*), which includes two genera—gooseberry *Grossularia Mill*. and currant *Ribes* L. Worldwide. There are about 200 species of the genus *Ribes* L., distributed mainly in the mountainous areas of Northern and Central Asia. More than 36 species are growing in the Commonwealth of Independent States (CIS) countries (Kazakhstan, Russia, Azerbaijan, Belarus, Armenia, Kyrgyzstan, Tajikistan, Turkmenistan, and Uzbekistan), and more than 11 species in Kazakhstan [2,3,4]. In recent decades, plants of the genus *Ribes* L. have attracted much attention due to the content of valuable biologically active substances with antioxidant, anti-inflammatory, antibacterial, antiviral and antitumor activity.

An analysis of the available literature revealed the most common species: *R. nigrum* L., *R. multiflorum* Poir., *R. uva-crispa* L., *R. biebersteinii* Berl., *R. aureum* Pursh, *R. alpinum* L., *R. meyeri* Maxim. and *R. rubrum* L. In medical practice, mainly the above-ground parts are used: leaves, fruits and stems of the *Ribes* L. species [5,6,7,8]. To date, more than 170 compounds have been isolated and identified from species of the genus *Ribes* L., which are classified as phenolic acids, flavonoids, anthocyanins, polysaccharides, procyanidins, organic acids, lignans, and fatty acids [9,10,11,12,13,14,15,16,17]. However, the phytochemical composition of many species of the genus *Ribes* L. has not been sufficiently studied, and therefore further in-depth research is required. This work aims to review and analyze the literature data on the study of species of the genus *Ribes* L., including their traditional use, classification of food components and their pharmacological activity.

## 2. Materials and Methods

Scientific information on plants of the genus *Ribes* L. published up to December 2024 was retrieved from online electronic databases such as Elsevier, Springer, Web of Science, PubMed, and Google Scholar. Specific search terms such (*Ribes* species OR *Ribes nigrum* OR blackcurrant OR gooseberry) AND (phylogeny OR phytochem* OR anthocyanin* OR polyphenol* OR antioxidant* OR Anti-Inflam* OR antimicrobial* OR biological activity OR clinical trial) were used as keywords to collect all relevant information. The information on the botanical description, geographical distribution, traditional use, phytochemistry, molecular phylogeny and pharmacological action of *Ribes* L. species was carefully selected and summarized. The summarized data were presented in tables within the full text of the article.

## 3. Results and Discussion

The initial search yielded a substantial number of 1282 articles. When a search was limited to *Ribes nigrum* OR blackcurrant, 1105 results appeared. After screening titles and abstracts and applying the inclusion and exclusion criteria, a total of 100 publications were selected for the final analysis. The main reasons for exclusion were irrelevance to the research topic, duplication, most reviews (113 articles), and lack of access to the full text. The results of this selection process form the basis for the subsequent sections, including the analysis of molecular phylogeny within the genus *Ribes*.

The *Grossulariaceae* family includes cultivated and wild berry bushes with 3- or 5-partite or single spines at the nodes of shoots, mostly with spiny internodes and alternate palmately lobed, serrated leaves. Flowers are bisexual, 5-membered, with a bell-shaped hypanthium, with oblong, outwardly recurved sepals and small petals, collected in 1–3-flowered tufted racemes, without peduncles. Column 1 is usually split no deeper than halfway; ovary stalks are long and generally many times longer than the ovary, not separating from the fruit. The fruit is a juicy berry with a dried flower at the top, like the ovary, smooth, hairy or glandular-bristly, prickly; seeds have a hard inner shell and a gelatinous outer shell [3].

Plants of the genus *Ribes* L. of the family *Grossulariaceae* are deciduous and evergreen shrubs, divided into sections: *Ribes*, *Grossularia* and *Parilla*. However, according to modern taxonomic classifications, the genus *Ribes* includes several additional sections, such as *Berisia*, *Calobotrya*, *Coreosma*, *Grossularioides*, *Heritiera*, *Hesperia*, *Lobbia*, *Robsonia*, and *Symphocalyx*, among others. This expanded classification reflects the morphological and molecular diversity within the genus. Species of this genus are morphologically characterized by bisexual or dioecious flowers collected in simple racemes. The calyx is bell-shaped or saucer-shaped, divided into five parts and connected to the ovary through a flat or tubular receptacle (hypanthium). The petals are attached to the base of the calyx, usually shorter than the calyx itself. Five stamens are located opposite the sepals and attached to the edge of the hypanthium or slightly below. The ovary is naked or covered with glandular hairs, two-celled and inferior, as well as, less often, semi-inferior. The fruits are juicy berries, which retain dried remains of the hypanthium and perianth at the top. Ripe berries are easily separated from the peduncle. The seeds have a hard inner shell and a gelatinous outer shell. Shrubs of this genus have alternate palmately lobed, serrated leaves without stipules [3].

Plants of the genus *Ribes* L. grow mainly in forest-steppe and subalpine zones of mountains, on rocky slopes, among shrubs and along rivers of the northern hemisphere [1,18,19,20]. Individual species *R. cysnobati* and *R. odoratum* H.L.Wendl. are found in the northeast, northwest and Central America [20]. According to research, the species *R. nigrum* Ben Sarek, *R. nigrum* Ometa, *R. nigrum* Triton and *R. nigrum* Tenah are widespread in Serbia [21]. In 2015, an Australian scientist Lim published his scientific paper on medicinal plants where the distribution of the hybrid *R. nigrum* and *R. uva crispa*, *R. x nidigrolaria* Rud. Bauer & A. Bauer in Italy was noted [22]. Rare currant species *R. magellanicum* Poir., *R. punctatum* Ruiz & Pav., *R. cucullatum* Hook. & Arn., *R. trilobum* Meyen., *R. valdivianum* Phil., and *R. gayanum* Reiche are found in Argentina and Chile [18,19,23]. Researchers from the Czech Tomas Bata University have studied some cultivated varieties of *R. uva-crispa*: *Invicta* (green gooseberry), *Rixanta* (green gooseberry), *Karat* (red gooseberry), *Black Negus* (red gooseberry), which mainly grow in England, Germany, the Czech Republic and Ukraine [24]. Chinese scientists have noted that the currant species *R. alpestre* Wall. ex Decne, *R. glaciale* Wall, *R. himalense* Royle ex Decne, *R. burejense* F. Schmidt, used in Tibetan medicine, were found in China [25]. Species *R. biebersteinii* Berl, *R. diacanthum* Pall and *R. orientale* Desf are common in Iran, Mongolia and Pakistan [5,10,26,27].

Extracts and juices from *R. nigrum* leaves are widely used in folk medicine to treat rheumatism, arthritis, and respiratory diseases. The plant accelerates wound healing and has pronounced diuretic and diaphoretic properties. It is also used for diarrhea and other gastrointestinal disorders [1,5,7,21,28]. In Mongolian folk medicine, an aqueous extract of *R. diacanthum* Pall. is used to treat urinary tract diseases [5,29,30]. Some medical studies have shown that the roots of *R. orientale* have long been used in India to treat rheumatism and joint pain [5,27,31,32].

According to M.S. Baitenov, in the flora of Kazakhstan there grow 11 currant species, each with a distinctive chemical composition. That makes them valuable from an ecological and pharmaceutical point of view [3]. Many currant species are of practical importance, primarily as sources of phenolic compounds. Species of the genus *Ribes* L. contain phenolic acids, flavonoids, anthocyanins, organic acids, and polysaccharides. Vitamins C and E have been found. Two species, *Ribes nigrum* and *R. turbinatum*, are endemic. *R. janczewskii* is listed in the Red Book of Kazakhstan. All Kazakhstan species of the genus *Ribes* L. were cultivated in the Main Botanical Garden (MBG) [3,4]. The currant *R. vulgare* Lam. is widespread in the foothill and mountainous regions of Kazakhstan. Its berries are rich in vitamins C and E, as well as phenolic compounds, which provide their antioxidant properties. The use of currant extracts in folk medicine is associated with its ability to strengthen the immune system and reduce inflammatory processes [4]. *R. hispidulum* (Jancz.) Pojark contains a significant amount of anthocyanins and vitamin C, and is used in folk medicine as an antipyretic, laxative, diaphoretic, haemostatic, diuretic, choleretic and anti-inflammatory remedy [4]. *R. altissimum* Turcz. ex Pojark., *R. atropurpureum* C. A. Mey, *R. Janczewskii* Pojark. * R. heterotrichum* C.A., *R. saxatile* Pallare, *R. meyeri* Maxim. fruits are used in folk medicine as a vitamin remedy [4].

Diverse authors have described the application of an infusion of *R. uva-crispa* leaves to relieve premenstrual syndrome pain and *R. alpestre* for joint pain [5,7,33,34,35,36]. The traditional use of blackcurrant as a diaphoretic and diuretic for diarrhea, spasmodic cough, and rheumatic pain is prescribed in the Herbal Society Monograph of the Herbal Medicines Committee in London (HMPC) [10,37]. The available studies do not provide sufficient information on the traditional use of other *Ribes* L. species.

### 3.1. Molecular Phylogeny of the Genus Ribes

Recent advances in molecular genetics have significantly enhanced our understanding of the taxonomy, phylogeny, and genetic diversity of the genus *Ribes* L. (*Grossulariaceae*). High-resolution approaches such as restriction site-associated DNA sequencing (RAD-seq), whole chloroplast genome sequencing, and analyses of nuclear ribosomal DNA (rDNA) have allowed for more precise elucidation of evolutionary relationships and the biogeographic history of *Ribes* species.

Comparative analyses of complete chloroplast genomes from *R. nigrum*, *R. rubrum*, and *R. uva-crispa* have identified highly polymorphic regions (e.g., ycf1, rpoC2) that are valuable for species delimitation and phylogenetic inference [38]. RAD-seq data further revealed genetic divergence and introgression among 30 species, resulting in the resolution of six well-defined subgeneric clades and demonstrating the evolutionary impact of hybridization events [39]. Additional phylogenetic studies based on nuclear ITS and chloroplast spacer regions (*trnL-trnF*, *psbA-trnH*) confirm the monophyly of subgenus *Grossularia* (gooseberries) and suggest its origin in North America, with subsequent radiation into East Asia and Europe [40]. Moreover, a study on the organellar genomes of *Ribes* species highlighted significant variability in chloroplast genes such as *ycf1*, *ycf2*, and *rpoC2*. Comparative analyses of mitochondrial genomes between *R. alpinum* and *R. nigrum* have identified mutation hotspots that may reflect a high level of genetic plasticity and adaptation, providing insights into the evolutionary dynamics [41].

Molecular marker development, particularly EST-SSRs and SNPs, has also enabled the construction of high-density genetic linkage maps for blackcurrant (*R. nigrum*), which play a crucial role in breeding programs aimed at developing stress-resistant cultivars [42].

To further clarify the systematic position of *R. meyeri*, we integrated this species into a broader phylogenetic framework. Molecular phylogenetic relationships within *Ribes* have been previously explored using chloroplast markers *(matK*, *rbcL*, *trnL-F*) and nuclear ITS regions, offering a robust basis for species-level classification and evolutionary analysis [43,44,45]. In our analysis, we combined published GenBank data with newly generated ITS and matK sequences for *R. meyeri*. Multiple sequence alignments were performed using MAFFT, and phylogenetic trees were constructed in MEGA X and MrBayes 3.2 under Bayesian Inference (BI) and Maximum Likelihood (ML) models, applying GTR + I + G substitution parameters as selected by jModelTest2.

**Figure 1:**
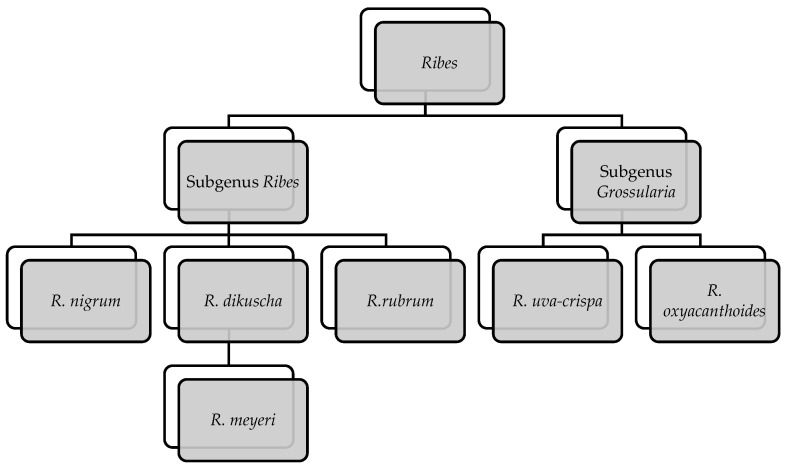
Phylogenetic relationships within the genus *Ribes*.

The resulting tree (Figure 1) shows two primary clades corresponding to subgenus *Ribes* (black and red currants) and subgenus *Grossularia* (gooseberries), consistent with previous large-scale phylogenetic analyses [45,46,47,48]. Within the *Ribes clade*, *R. meyeri* clusters closely with *R. nigrum*, *R. dikuscha*, and *R. rubrum*, forming a strongly supported subgroup of Eurasian black currants (posterior probability = 0.97; bootstrap = 91). This result aligns with previous hypotheses based on morphological traits such as leaf venation, glandular trichomes, and fruit pigmentation [44].

The inclusion of *R. meyeri* in molecular analyses provides valuable insight into the genetic structure and evolutionary position of this species. Its close phylogenetic proximity to *R. nigrum*, a commercially and medicinally important plant, suggests the potential for similar profiles of bioactive compounds. This finding highlights the importance of further phytochemical and pharmacological studies targeting *R. meyeri.*

Tree topology based on combined nuclear (ITS) and chloroplast (*matK*, *rbcL*, *trnL-F*) markers, reconstructed using Bayesian inference and Maximum Likelihood methods. Posterior probabilities and bootstrap values are shown at the nodes.

### 3.2. Phytochemical Study of the Genus Ribes L.

Through the study of literature and scientific sources, species of the genus *Ribes* L. were determined to be a rich source of phenolic compounds: flavonoids, proanthocyanidins and hydroxycinnamic acids. Over the past few decades, there have been many studies on the isolation and identification of biologically active compounds in plants of this genus. Flavonoids, organic acids, aromatic components, essential oils and polysaccharides were found in all vegetative organs of plants of the genus *Ribes* L., and their potential pharmacological activity was also established.

To study the phytochemical composition of plants of the genus *Ribes* L., various quantitative and qualitative analyses from different countries were reviewed. The plants of this genus are a rich source of phenolic compounds. Various world scientists obtained 22 flavonoids from different parts of the *R. nigrum* plant (juices and extracts from berries and leaves) [5,49,50,51,52,53,54,55,56,57]. Derivatives of kaempferol and quercetin were found in the leaves of plants of the genus *Ribes* L. [58]. The aglycone structure of flavonoids is represented by aurones (e.g., aureusidin), flavonols such as quercetin, myricetin-3-O-glucoside, myricetin-3-O-galactoside, myricetin-3-O-rutinoside, quercetin-3-O-rutinoside, epigallocatechin, and anthocyanidins. The sugar moiety is most commonly glucose and rutinose, although galactose (e.g., in myricetin-3-O-galactoside) and arabinose are also occasionally present [49,50,51,52,53,54,55]. Phenolic compounds isolated from *R. nigrum* species are listed in Table 1.

A comparative mass-spectrometric study by Razgonowa et al. profiled four understudied species (*R. pauciflorum* Turcz., *R. triste* Pall., *R. dicuscha* Fisch., and *R. aureum* Purch.), identifying diverse polyphenols and bioactive compounds [59]. This work maps the unique phytochemical landscapes across these species—foundational for targeted biological activity studies. The newly identified polyphenols include flavones, flavonols, flavan-3-ols, lignans, coumarins, stilbenes, and others. The other freshly detected compounds in *Ribes* species include anthraquinone derivatives (such as 1,8-dihydroxy-anthraquinone and 1,3,6,8-tetrahydroxy-9(10H)-anthracenone), naphthoquinones (e.g., 8,8′-dihydroxy-2,2′-binaphthalene-1,1′,4,4′-tetrone), polyhydroxycarboxylic acids, omega-3 fatty acids (stearidonic acid, linol enic acid), and others.

#### 3.2.1. Organic Acids

Organic acids in plants of the genus *Ribes* L. play an important role in their biochemical composition, significantly affect their taste properties and have antioxidant properties [68]. From the species of the genus *Ribes* L., 27 organic acids have been isolated, including groups of acids (phenolic, tannic and fatty) and citric, malic, oxalic, succinic and salicylic acids (Table 1).

According to the studies, blackcurrant juice contains p-coumaric, ferulic, caffeic, p-hydroxybenzoic, ellagic acid and gallic acid monohydrate. A total of 19 phenolic acids were isolated from blackcurrant leaves, including coumaric, o-coumaric, ferulic, isoferulic, ellagic, sinapic, caffeic, chlorogenic, neochlorogenic, cryptochlorogenic, salicylic, protocatechuic, dihydroxyphenylacetic, 2,5-dihydroxybenzoic, vanillic, gallic, syringic, p-hydroxybenzoic and 4-hydroxyphenylacetic acids [5,64].

Fatty acids such as linoleic, stearidonic, oleic, palmitic and stearic acids, which are also detected in *Ribes* species, are presented in Table 1.

#### 3.2.2. Volatile Compounds

Recently, the essential oil from blackcurrant buds (*R. nigrum*) is used mainly as a valuable perfume ingredient. American scientists presented a complete characterization of dormant buds of different blackcurrant cultivars (*R. nigrum*) grown in Northern European countries. Essential oils were isolated from the buds by hydrodistillation and analyzed with gas chromatography-mass spectrometry (GC-MS), GC-flame ionization detection (GC-FID) and GC-olfactometry (GC-O). The most common compounds in the essential oil of blackcurrant buds were sabinene, δ-3-carene, and terpinolene [68,69,70].

### 3.3. Biological Activity

Studies have been conducted to assess the pharmacological effects of biologically active substances obtained from representatives of the genus *Ribes* L. based on different biological models—in vivo, in vitro and in situ [5]. Extracts and secondary metabolites isolated from various species of the genus *Ribes* L. demonstrate a wide range of biological properties: antihyperlipidemic, antioxidant, antibacterial, antitumor, antiviral, neuroprotective and other properties [5]. Scientists indicated that blackcurrant polysaccharides can bind with specific enzymes in the human body, further inhibiting their activities and preventing pathological conditions. These polysaccharides showed potent antioxidant, anti-inflammatory activities and protective effects on erythrocyte [66]. In addition, they effectively inhibited the activities of α-amylase, α-glucosidase, and acetylcholinesterase, and thereby displayed the potential to mitigate hyperglycemia [71] and prevent Alzheimer’s disease [72]. Moreover, Huang et al. isolated two kinds of acid heteropolysaccharides (PRNP-1 and PRNP-2) from *R. nigrum* L. PRNPs markedly decreased the serum concentration of uric acid and creatinine, and xanthine oxidase (XOD) activity in hyperuricemia mice model. Therefore, PRNPs may be the potential natural therapeutic agents for gout treatment [73].

Extracts from *R. magellanicum* fruits exhibited potent inhibitory effects on carbohydrate-metabolizing enzymes, particularly α-glucosidase, with IC_50_ values ranging from 0.06 to 0.29 μg/mL. This suggests potential applications in managing postprandial hyperglycemia [74]. Also, Lappi et al. [75], in a randomized crossover trial, showed that 75 g of blackcurrant and the product with fermented quinoa were able to lower postprandial glycaemia and insulinaemia. Moreover, randomized clinical trial from Iran, assessing the antihyperglycemic and hypolipidemic effects of hydro-ethanolic extract of *Ribes khorassanicum*, showed that co-supplementation of diabetic patients with *R. khorasanicum* extract ameliorated hyperglycemia and hyperlipidemia without causing any adverse effects [76].

*Ribes nigrum* L. is a berry rich in anthocyanins, bioactive compounds known for their antioxidant and neuroprotective properties that benefit human health. Behavioral tests conducted by da Costa et al. [77] revealed that blackcurrant and/or Donepezil prevented the learning and memory deficits induced by Scopolamine in adult Swiss mice. Results suggest that blackcurrant and Donepezil, either alone or in combination, have anti-amnesic effects by modulating cholinergic system enzymes and improving the redox profile. They concluded that blackcurrants could be used as a natural supplement for the prevention and treatment of memory impairment in neurodegenerative diseases [77]. Shimada et al. [78] demonstrated that anthocyanin-rich blackcurrant extract improves the long-term recognition memory impairment and emotional abnormality of SAMP8 mice, a mouse model characterized by several pathological features of Alzheimer’s disease. Real-time PCR verified alterations in the expression of Alzheimer’s disease-related genes. These findings indicate that anthocyanin-rich blackcurrant extract may be a useful food supplement or ingredient for the prevention of Alzheimer’s disease.

Sarmentosin, a glycoside identified in *R. nigrum* (blackcurrant), has been shown to inhibit human platelet monoamine oxidase (MAO), indicating potential benefits in mood regulation and cardiovascular health. Additionally, it modulates the Nrf2 pathway, enhancing mitophagy and reducing oxidative stress, which may offer protection against acetaminophen-induced liver injury [79,80].

*Ribes* seed extracts of some unexplored taxa belonging to the *Ribes* genus were shown as potentially raw sources of healthy phenolic compound-rich seed oils, in addition to their already known GLA-rich FA profiles. The growth inhibition against HT-29 cancer cells was tested, demonstrating a correlation between phenolic profiles and cytotoxicity toward colorectal cancer cell lines, suggesting their antitumor potential [81].

Nosal et al. [82] investigated the effects of blackcurrant on gut microbiota abundance and composition, inflammatory and immune responses, and their relationship with bone mass changes. Evidence from a pilot randomized controlled trial revealed that daily blackcurrant consumption for 6 months mitigated bone loss in this population, potentially through modulating the gut microbiota composition and suppressing osteoclastogenic cytokines [82].

The acute effects of an anthocyanin-rich blackcurrant beverage, compared with a matched placebo, on selected markers of cardiovascular disease risk in healthy middle-aged subjects in response to a high-fat meal were investigated in a randomized, double-blind, placebo-controlled, crossover trial [83]. The trial results indicated that a blackcurrant beverage, rich in anthocyanin, mitigated the effects of a high-fat meal on vascular function and markers of cardiovascular risk, and this was associated with the appearance of specific plasma anthocyanin phenolic metabolites.

#### 3.3.1. Antimicrobial Activity

German scientists isolated polysaccharides from blackcurrant seeds (*R. nigrum* L.) and analyzed their effect against *Helicobacter pylori* on sections of human gastric mucosa in situ. After preliminary treatment of *H. pylori* with 0.01–0.1% solutions of the isolated polysaccharide (RPS), bacteria sticking to the epithelium were significantly reduced depending on the concentration compared to the untreated control suspension [84]. The antifungal activity of *Ribes* L. species extracts was studied at the Department of Microbiology, University of Szeged, Hungary. It was found that the growth of most *Candida* species was inhibited (minimal inhibitory concentration (MIC) values ranged from 2.82 to 10.98 mg/mL dry matter content), except for *C. albicans*, *C. krusei*, *C. lusitaniae* and *C. pulcherrima*, and that there was a significant dose-effectiveness relationship between the antifungal activity and the phenolic content of *Ribes* L. species extracts [5,85].

The authors examined the fruits of four species of the genus *Ribes* L., their phenolic composition, and the antimicrobial activity of the extracts. They used spectrophotometric analysis and high-performance liquid chromatography (HPLC) to quantify the main bioactive compounds. The extracts demonstrated significant antimicrobial activity against several pathogens, including *Escherichia coli*, *Staphylococcus aureus*, and *Candida albicans*. It was found that samples with a lower ratio of anthocyanins to total phenolic content had a more pronounced antimicrobial effect. This indicates that, along with anthocyanins, other phenolic compounds such as flavonoids and proanthocyanidins play an important role in the biological activity of *Ribes* L. plant extracts. This study highlights the potential of *Ribes* L. fruits as natural sources of antimicrobial compounds, which may be important for new pharmaceutical and food antimicrobial agents production [86].

The antibacterial activity of plant extracts is due to the presence of phenolic compounds in the genus *Ribes* L. plants [4]. Positive correlations have been found between the content of phenolic compounds and the antibacterial activity of extracts of species of the genus *Ribes L*. against *S. aureus* and *Bacillus cereus*. It has been established that the antibacterial capacity of phenolic acids depends mainly on the presence of carboxyl groups and the substitution pattern in the benzene ring, as well as on the number of hydroxyl groups in the molecules [86].

*R. nigrum* extracts were used to green-synthesize silver nanoparticles (AgNPs), which showed potent antibacterial activity, likely attributable to their small size and spherical morphology. Compounds presented in the extract might also act as natural stabilizers, contributing to the enhanced stability and extended shelf life of the nanoparticles. The authors proposed that these biogenic AgNPs exert their antibacterial effects by disrupting membrane permeability, potentially through modulation of H^+^-translocating ATPase activity, interference with energy-dependent proton fluxes, and alteration of the formate hydrogenlyase (FHL) proton–potassium transport system [87].

#### 3.3.2. Antiviral Activity

Haasbach et al. investigated the antiviral potential of wild blackcurrant (*Ribes nigrum*) leaf extract Ladania067 against the influenza A virus. According to the results, the extract was non-cytotoxic in several cell lines and did not affect human lymphocyte proliferation at concentrations up to 1 mg/mL. Notably, Ladania067 demonstrated high efficacy against the pandemic influenza A/H1N1 strain. Mechanistic studies showed that the extract blocked the early stages of the viral cycle, especially when added immediately after infection. In mice, intranasal administration of 500 μg Ladania067 reduced by 85% viral titers in the lungs 24 h after infection. These data indicate the potential of using blackcurrant leaf extract as a source of new antiviral agents against the influenza *A virus* [56].

#### 3.3.3. Regenerative Activity

Turkish scientists conducted a comprehensive study of the therapeutic activity of secondary metabolites of species of the *Ribes* L. genus. Particular attention was paid to black currant (*Ribes nigrum*), used in folk medicine to treat skin lesions and accelerate wound healing. The study assessed the antioxidant, anti-inflammatory, and wound-healing activities of extracts from the raw materials of various *Ribes* L. species. In vitro and in vivo experiments have demonstrated that blackcurrant extracts significantly stimulate healing processes, as evidenced by enhanced tissue regeneration and reduced inflammation at the site of injury. The main biologically active components of the extracts, such as flavonoids and anthocyanins, exhibit a pronounced antioxidant and anti-inflammatory effect, contributing to accelerated tissue recovery. The study results confirm the traditional use of blackcurrant, and also show the promise of using extracts of *Ribes* L. species, including *Ribes nigrum* extract, in the development of agents for the treatment of wounds and acceleration of tissue regeneration [7].

#### 3.3.4. Antioxidant Activity

Several studies have shown that antioxidant and antitumor activity are closely related to the content of phenolic compounds. There are many methods for determining antioxidant activity, but the most commonly used is the DPPH method [88,89]. Extracts from young blackcurrant leaves showed the highest antioxidant activity compared to the extract from leaves collected at later stages of growth [5]. Scientists from the Romanian University of Medicine and Pharmacy determined the antioxidant activity of the extracts by the ability to inhibit free radicals of 1,1-diphenyl-2-picrylhydrazyl (DPPH) and 2,2′-azino-bis(3-ethylbenzothiazoline-6-sulfonic acid) (ABTS), and by photochemiluminescence (PCL) as well. Identification and quantification of individual phenolic compounds were performed using a high-performance liquid chromatograph with a diode array detector (HPLC-DAD). Methanol and methanol 50% extract showed the highest antioxidant activity for blackcurrant. The antioxidant activity, presented as a concentration of a substance required to inhibit a biological or biochemical function by 50% (IC_50_) of the methanol extract was 336.53 ± 7.65 μg/mL for DPPH and 210.65 ± 12.51 μg/mL for ABTS, while the water extract showed IC_50_ values of 554.32 ± 13.69 μg/mL (DPPH) and 248.07 ± 12.46 μg/mL (ABTS). For comparison, the ethyl acetate extract exhibited the highest antioxidant activity, with IC_50_ values of 194.68 ± 9.84 μg/mL (DPPH) and 104.14 ± 2.39 μg/mL (ABTS). In blackcurrant, the main compound was cyanidin-3-glucoside. Quercetin-3-O-glucoside was identified in every sample. It was a neochlorogenic acid of the cinnamic acid derivatives, which was present in blackcurrant in the highest amount (356.33 μg/g) [57].

Zhao et al. (2021) [60] conducted a qualitative and quantitative analysis of phenolic compounds in *Ribes meyeri* leaves using HPLC-QTOF-MS/MS and UHPLC-MS/MS. The authors identified 77 phenolic compounds, including rutin, epigallocatechin, isoquercetin, epicatechin, protocatechuic acid, and kaempferol-3-O-rutinoside, which were present in significant amounts. The methanol extract and four different fractions showed the ability to enhance glucose uptake in 3T3-L1 adipocytes, indicating their potential antidiabetic activity. In addition, the ethyl acetate fraction showed high contents of total phenolics (966.89 ± 3.59 mg gallic acid/gram) and flavonoids (263.58 ± 17.09 mg catechin/gram) and significant inhibitory activity against protein tyrosine phosphatase 1B (IC50: 0.60 ± 0.03 μg/mL). This study provides the first detailed analysis of the phenolic composition of *R. meyeri* leaves and confirms their antioxidant activity and antidiabetic properties [60]. Also, *R. meyeri* fruits may play a hypoglycemic role in different targets. The results revealed that cyanidin-3-O-rutinoside was the predominant anthocyanin in *R. meyeri* fruits. The anthocyanin-rich fraction demonstrated notable inhibitory effects on α-amylase and α-glucosidase, and significantly enhanced glucose uptake in 3T3-L1 adipocytes [62].

A comprehensive study employed chromatographic techniques to isolate four quercetin derivatives from *R. himalense*. These compounds demonstrated significant free-radical-scavenging activity. Molecular docking analyses revealed strong binding affinities to several oxidative stress-related proteins, including acetylcholinesterase (AChE), NADPH-oxidase, and xanthine oxidase (XOD), suggesting potential therapeutic applications in neurodegenerative and inflammatory conditions [63].

Flavonoids are very promising in anti-aging research. In the study conducted by Gao et al. [90], *R. meyeri* anthocyanin extract was analyzed for its effects on neural stem cell (NSC) senescence in vivo and in vitro. The findings suggested that *R. meyeri* anthocyanins increase NSC proliferation and improve neurogenesis with aging via Nar-induced reductions in TNF-α protein levels in vivo [90]. Black currants (*Ribes nigrum* L.), known as a “super fruit” due to the alleviation of oxidative stress-related disorders, was investigated for the protective effects in UVB-irradiated human dermal fibroblasts (NHDFs). Treatment with *R. nigrum* in UVB-irradiated skin models alleviated UVB-mediated photoaging and improved the expression of type I procollagen [58].

#### 3.3.5. Anti-Inflammatory Activity

Ivanova D.A. et al. provided data on the anti-inflammatory activity of various plants, including species of the genus *Ribes* L. The article indicates that extracts from species of the genus *Ribes* L., *R. nigrum* in particular, demonstrated pronounced anti-inflammatory activity in both in vitro and in vivo models. Research data showed that plant extracts suppress inflammatory reactions, which makes them promising for creating herbal drugs to treat inflammatory diseases. The active components of plants of the genus *Ribes* L. reduce inflammation by acting on key inflammatory molecules and modulating immune activity. The work emphasizes the need for further research to more closely understand the mechanisms of action and the possibility of using *Ribes* L. extracts in clinical practice to treat inflammatory disorders [91].

Research of Gulmez at al. (2022) revealed that *Ribes rubrum* fruit extract exerted antioxidant, anti-inflammatory, and antithrombotic effects in streptozotocin-induced diabetic rats, reducing TNF-α, normalizing platelet P-selectin, and protecting mitochondrial integrity which may be beneficial in preventing diabetic complications [92].

Recently published study investigated *Ribes nigrum* leaf extract in microglial (brain immune) cells. It showed downregulation of pro-inflammatory gene expression and restoration of redox balance, suggesting potential for neuroprotective or anti-neuroinflammatory therapies. The extract was shown to modulate the activity of key enzymes involved in oxidative stress regulation, including catalase and acyl-CoA oxidase 1 (ACOX1), as well as the expression of related genes such as Cat, iNos, Il-1β, Tnf-α, and Abcd1 in both wild-type (Wt) and Acox1^−^/^−^ BV-2 cells. Prolonged exposure led to a decrease in catalase activity and a corresponding increase in ACOX1 activity. Additionally, the extract significantly reduced intracellular reactive oxygen species (ROS) and nitric oxide (NO) levels, highlighting its strong antioxidant capacity in the tested microglial cell models [93].

Other recent studies also support the anti-inflammatory potential of *Ribes* species. For instance, Magnavacca et al. (2021) demonstrated that leaf extract of *R. nigrum* selectively inhibited IFN-γ–induced pro-inflammatory cascades in HaCaT keratinocytes, decreasing IL-6, IL-8, sICAM-1, and TSLP secretion [94]. Similarly, Garbacki et al. (2004) showed that proanthocyanidins from *R. nigrum* leaves reduced carrageenan-induced paw edema and pleurisy in rats, with significant suppression of leukocyte infiltration and nitric oxide overproduction [95]. Furthermore, Karlsen et al. (2007) reported that anthocyanin-rich extracts from blackcurrant and blueberry inhibited NF-κB activation in monocytes and reduced plasma concentrations of pro-inflammatory mediators in healthy volunteers, providing clinical evidence of systemic anti-inflammatory effects [96]. It was demonstrated that polyphenol-rich blackcurrant extract prevented inflammation in the liver and adipose tissue of diet-induced obesity (DIO) mice [97,98]. Furthermore, the same researchers have shown increases in the hepatic expression of M1 macrophage markers, which was significantly attenuated by blackcurrant in DIO mice [99] as well as they demonstrated that blackcurrant extract suppresses M1 polarization of macrophages, leading to repressed pro-inflammatory responses, and suggesting that metabolites of blackcurrant may not exert the anti-inflammatory effect of blackcurrant directly by altering macrophage phenotypes, but it may attenuate inflammatory responses in macrophages by modulating levels of obesity-induced circulating pro-inflammatory factors [100].

Taken together, these findings suggest that *Ribes* species exert their anti-inflammatory action through multiple mechanisms, including suppression of NF-κB activation, inhibition of cytokine and chemokine release, reduction in nitric oxide production, and modulation of leukocyte migration. This multimodal activity positions *Ribes* extracts as promising candidates for phytotherapeutic interventions in inflammatory and immune-related disorders.

## 4. Conclusions

It is well established that plants of the genus *Ribes* L. contain a rich variety of phenolic compounds, which contribute to their biological activity. To date, extracted and identified compounds from plants of the genus *Ribes* L. belong to various classes: flavonoids, phenolic acids, essential oils, fatty acids, aliphatic hydrocarbons and aldehydes. They have shown extensive pharmacological activity.

The mechanisms of action of extracts and individual compounds isolated from the genus *Ribes* L. plants have now been elucidated. Pharmacological studies In Vivo and In Vitro have confirmed that they exhibit important biological activity and have antihyperlipidemic, antioxidant, anti-inflammatory, antiviral, antibacterial, antimicrobial and anticancer effects. Medicines from the raw materials of plants of this genus, being powerful natural antioxidants, can be used as an alternative to synthetic medications.

Numerous studies have demonstrated that *Ribes* species contain a wide range of bioactive compounds, including flavonols, anthocyanins, and phenolic acids, which contribute to their antioxidant, anti-inflammatory, and antidiabetic properties. These findings support the view that *Ribes* spp. are not only well-known sources of phenolic compounds, but also promising candidates for further research aimed at developing new herbal preparations for the prevention and treatment of chronic diseases. However, more in-depth phytochemical studies, studying the full potential of the isolated secondary metabolites, evidence of the safety and effectiveness of plant extracts or isolated pure compounds, studies of the mechanisms of action, pharmacokinetics, and clinical trials are necessary. Since published In Vivo and non-clinical studies on various extracts of the *Ribes* L. genus are extremely scarce, toxicity studies are essential before developing any pharmaceutical formulations to avoid potentially harmful effects on human health. In-depth studies are also needed to clarify information on the traditional use of *Ribes* L. plants. Recent studies have significantly expanded our understanding of *Ribes* spp., especially in immunomodulation and neuroprotection, cancer-related cytotoxicity, metabolic health applications (antidiabetic, antioxidant) and innovative uses in nanotechnology. Numerous studies have confirmed the chemical composition and biological activity of secondary metabolites in black currant (*Ribes nigrum*). The Kazakhstani species of the genus Currant *Ribes* L. are used only in folk medicine, and this opens up prospects, providing an opportunity to conduct further research. The solution to these problems will be foremost for discovering the sources of biologically active compounds of the genus *Ribes* L. plants.

## Figures and Tables

**Table 1:** Phytochemical content of *Ribes* spp.

Chemical Group	Compounds	Species	Source
Aurones	Aureusidin	*R. nigrum*	[52,53,54]
Flavanones	Pinocembrine 7-methyl ether	*R. viscossisimum*	[14]
Flavones	Formononetin	*R. aureum*	[59]
	Luteolin-O-hexoside	*R. aureum*, *R. triste*, *R. pauciflorum*	[59]
	Acacetin	*R. triste*	[59]
	Dihydroxy-methoxy(iso)-flavone	*R. aureum*, *R. triste*, *R. pauciflorum*, *R. dikuscha*	[59]
	Cirsimaritin	*R. aureum*, *R. triste*, *R. pauciflorum*, *R. dikuscha*	[59]
	Dihydroxy-dimethoxy(iso)flavone	*R. aureum*, *R. triste*, *R. pauciflorum*, *R. dikuscha*	[59]
	Chrysoeriol 7-O-neohesperidoside	*R. aureum*, *R. triste*, *R. pauciflorum*, *R. dikuscha*	[59]
	Chrysoeriol O-rhamnosyl glucoside	*R. aureum*, *R. triste*, *R. pauciflorum*, *R. dikuscha*	[59]
	Isovitexin	*R. aureum*	[59]
	Tetrahydroxydimethoxyflavone	*R. dikuscha*	[59]
	Luteolin	*R. meyeri*, *R. triste*	[59,60]
	Apigenin	*R. meyeri*, *R. triste*, *R. pauciflorum*, *R. aureum*	[59,60]
	Hydroxygenkwanin	*R. meyeri*	[60]
Flavonols	Galangin	*R. viscossisimum*	[14]
	Myricetin	*R. nigrum*	[49,54]
	Kaempferol	*R. nigrum*, *R. mandshuricum*, *R. pauciflorum*, *R. dikuscha*, *R. triste*, *R. meyeri*	[55,58,59,60,61]
	Quercetin	*R. nigrum*, *R. meyeri*, *R. triste*, *R. pauciflorum*, *R. dikuscha*	[55,59,60]
	Myricetin-3-O-glucoside	*R. nigrum*	[54]
	Myricetin-3-O-galactoside	*R. nigrum*	[54]
	Myricetin-3-O-arabinoside	*R. nigrum*	[54]
	Myricetin-3-O-rutinoside	*R. nigrum*	[54]
	Dihydroquercetin	*R. nigrum*	[54]
	Dihydromyricetin	*R. meyeri*	[62]
	Quercetin-3-O-glucoside	*R. nigrum*, *R.meyeri*	[54,62]
	Quercetin-3-O-galactoside	*R. nigrum*	[55]
	Quercetin-3-O-arabinoside	*R. nigrum*	[54]
	Quercetin-3-O-rutinoside	*R. nigrum*	[57]
	Kaempferol-3-O-glucoside	*R. nigrum*	[55,57]
	Kaempferol-3-O-rutinoside	*R. nigrum*, *R. meyeri*	[56,60]
	Rutin	*R. nigrum*, *R. meyeri*	[56,60]
	Hyperoside	*R. nigrum*	[56]
	Myricetin 3-O-glucoside	*R. nigrum*	[55]
	Isoquercitrin	*R. meyeri*	[60]
	Astragalin	*R. meyeri*, *R. pauciflorum*, *R. dikuscha*	[59,60]
Flavan-3-ols	Epicatechin	*R. meyeri*	[60]
	Epigallocatechin	*R. nigrum*, *R. meyeri*	[55,60]
Anthocyanins	Cyanidin-3-O-glucoside	*R. nigrum*, *R. meyeri*	[56,62]
	Pelargonidin	*R. nigrum*, *R. meyeri*	[51,62]
	Pelargonidin3-O-glucoside	*R. nigrum*	[51]
	Pelargonidin3-O-rutinoside	*R. nigrum*	[51]
	Peonidin	*R. nigrum*	[51]
	Peonidin3-O-glucoside	*R. nigrum*	[51]
	Peonidin3-O-rutinoside	*R. nigrum*	[51]
	Cyanidin	*R. nigrum*	[51]
	Delphinidin	*R. nigrum*	[51]
	Petunidin Chloride	*R. nigrum*	[51]
	Malvidin	*R. nigrum*	[51]
	Malvidin3-O-glucoside	*R. nigrum*	[51]
	Cyanidin3-O-arabinoside	*R. nigrum*	[51]
	Cyanidin-3-O-rutinoside	*R. nigrum*, *R. meyeri*	[51,62]
	Delphinidin-3-O-rutinoside	*R. nigrum*, *R. meyeri*	[51,62]
	Delphinidin-3-O-glucoside	*R. nigrum*, *R. meyeri*	[51,63]
	Delphinidin-3,5-dihexoside	*R. dikuscha*	[59]
	Petunidin-3-O-glucoside	*R. dikuscha*	[59]
Phenolic acids	*p*- Coumaric acid	*R. nigrum*, *R. meyeri*	[62,64]
	*o*- Coumaric acid	*R. nigrum*	[65]
	Ferulic acid	*R. nigrum*	[66]
	Isoferulinic acid	*R. nigrum*	[65]
	Gallic acid monohydrate	*R. nigrum*	[65]
	*p*-Hydroxybenzoic acid	*R. nigrum*	[65]
	Ellagic acid	*R. nigrum*	[65]
	Sinapic acid	*R. nigrum*	[55]
	Caffeic acid	*R. nigrum*, *R. meyeri*	[60,62,65]
	Chlorogenic acid	*R. nigrum*	[65]
	Neochlorogenic acid	*R. nigrum*	[55]
	Cryptochlorogenic acid	*R. nigrum*	[55]
	Salicylic acid	*R. nigrum*	[65]
	Protocatechuic acid	*R. nigrum*, *R. mandshuricum*, *R. meyeri*	[58,60,65,67]
	Dihydroxyphenylacetic acid	*R. nigrum*	[65]
	2.5-dihydroxybenzoic acid	*R. nigrum*	[65]
	Vanillic acid	*R. nigrum*	[65]
	Gallic acid	*R. nigrum*, *R. mandshuricum*	[19,20,21,55,61]
	Syringic acid	*R. nigrum*, *R. diacanthum* Pall.	[65,67]
	*p*-Hydroxybenzoic acid	*R. nigrum*	[65]
	4-Hydroxyphenylacetic acid	*R. nigrum*	[65]
	Tannins acids	*R. nigrum*	[21]
Fatty acids	Linoleic acid	*R. nigrum*	[21]
	Stearidonic acid	*R. nigrum*	[21]
	Oleic acid	*R. nigrum*	[21]
	Palmitic acid	*R. nigrum*	[21]
	Stearic acid	*R. nigrum*	[21]

## Data Availability

All data supporting the findings of this study are available in the published literature and publicly accessible sources cited in the manuscript.

## Abbreviations

The following abbreviations are used in this manuscript:

WHO	Worls Health Organizaton
HMPC	Committee on Herbal Medicinal Products
DNA	Deoxyribonucleic Acid
RAD-seq	Restriction Site-Associated DNA Sequencing
ITS	Internal Transcribed Spacer
MAFFT	Multiple Alignment using Fast Fourier Transform
BI	Bayesian Inference
ML	Maximum Likelihood
GC-MS	Gas Chromatography-Mass Spectrometry
GC-FID	GC-flame ionization detection
GC-O	GC-olfactometry
AE	Acetone
SDWF	Spray-Dried Water Fraction
FDWF	Freeze-Dried Water Fraction
ME	Methanol
RAC	Radical Absorption Capacity
DPPH	2,2-Diphenyl-1-picrylhydrazyl
ABTS	2,2-Azino-bis(3-ethylbenzothiazoline-6-sulfonic acid)
PRNPs	Plant Related Natural Products
XOD	Xanthine Oxidase
MAO	Monoamine Oxidase
IC_50_	Half Maximal Inhibitory Concentration
MIC	Minimum Inhibitory Concentration
HPLC	High-Performance Liquid Chromatography
AgNPs	Silver Nanoparticles
FHL	Formate Hydrogenlyase
HPLC-DAD	High-Performance Liquid Chromatograph with a Diode Array Detector
HPLC-QTOF-MS/MS	High-Performance Liquid Chromatography coupled with Quadrupole Time-of-Flight Tandem Mass Spectrometry
UHPLC-MS/MS	Ultra-High-Performance Liquid Chromatography coupled with Tandem Mass Spectrometry
AChE	Acetylcholinesterase
ABG	Altai Botanical Garden
MBG	Main Botanical Garden

## References

[1] Zdunić G., Šavikin K., Pljevljakušić D., Djordjević B. (2016). Black (*Ribes nigrum* L.) and Red Currant (*Ribes rubrum* L.) Cultivars. Nutritional Composition of Fruit Cultivars.

[2] Cortez R.E., Gonzalez De Mejia E. (2019). Blackcurrants (*Ribes nigrum*): A Review on Chemistry, Processing, and Health Benefits. J. Food Sci..

[3] Academy of Sciences of the Kazakh SSR (1961). Flora of Kazakhstan.

[4] Grudzinskaya L.M., Gemedzhieva N.G., Nelina N.V., Karzhaubekova Z.Z. (2014). Annotated List of Medicinal Plants of Kazakhstan: Reference Publication.

[5] Sun Q., Wang N., Xu W., Zhou H. (2021). Genus Ribes Linn. (Grossulariaceae): A comprehensive review of traditional uses, phytochemistry, pharmacology and clinical applications. J. Ethnopharmacol..

[6] Gopalan A., Reuben S.C., Ahmed S., Darvesh A.S., Hohmann J., Bishayee A. (2012). The health benefits of blackcurrants. Food Funct..

[7] Kendir G., Süntar I., Çeribaşı A.O., Köroğlu A. (2019). Activity evaluation on Ribes species, traditionally used to speed up healing of wounds: With special focus on Ribes nigrum. J. Ethnopharmacol..

[8] Shaw O.M., Nyanhanda T., McGhie T.K., Harper J.L., Hurst R.D. (2017). Blackcurrant anthocyanins modulate CCL11 secretion and suppress allergic airway inflammation. Mol. Nutr. Food Res..

[9] Cyboran S., Bonarska-Kujawa D., Pruchnik H., Żyłka R., Oszmiański J., Kleszczyńska H. (2014). Phenolic content and biological activity of extracts of blackcurrant fruit and leaves. Food Res. Int..

[10] Delazar A., Khodaie L., Afshar J., Nahar L., Sarker S. (2010). Isolation and free-radical-scavenging properties of cyanidin 3-O-glycosides from the fruits of *Ribes biebersteinii* Berl. Acta Pharm..

[11] Dobson G. (2000). Leaf lipids of *Ribes nigrum*: A plant containing 16:3, α-18:3, γ-18:3 and 18:4 fatty acids. Biochem. Soc. Trans..

[12] Kendir G., Köroğlu A. (2015). In vitro Antioxidant Effect of the Leaf and Branch Extracts of *Ribes* L. Species in Turkey. Int. J. Pharm. Sci..

[13] Kiliç C.S., Koyuncu M., Özek T., Başer K.H.C. (2008). Essential Oil of the Leaves of *Ribes nigrum* L. from Turkey. J. Essent. Oil Res..

[14] Paunović S.M., Mašković P., Nikolić M., Miletić R. (2017). Bioactive compounds and antimicrobial activity of black currant (*Ribes nigrum* L.) berries and leaves extract obtained by different soil management system. Sci. Hortic..

[15] Sasaki T., Li W., Zaike S., Asada Y., Li Q., Ma F., Zhang Q., Koike K. (2013). Antioxidant lignoids from leaves of *Ribes nigrum*. Phytochemistry.

[16] Tabart J., Kevers C., Evers D., Dommes J. (2011). Ascorbic Acid, Phenolic Acid, Flavonoid, and Carotenoid Profiles of Selected Extracts from Ribes nigrum. J. Agric. Food Chem..

[17] Tian Y., Liimatainen J., Alanne A.-L., Lindstedt A., Liu P., Sinkkonen J., Kallio H., Yang B. (2017). Phenolic compounds extracted by acidic aqueous ethanol from berries and leaves of different berry plants. Food Chem..

[18] Banados M.P., Hojas C., Patillo C., Gonzales J. (2002). Geographical distribution of native ribes species present in the herbarium of Chile. Acta Hortic..

[19] Hoffmann J.A., Jullián A. (2005). Flora Silvestre de Chile: Zona Araucana; Una Guía Ilustrada para la Identificación de las Especies de Plantas le~Nosas del sur de Chile (entre el río Maule y el seno de Reloncaví); [Arboles, Arbustos y Enredaderas le~Nosas].

[20] Wilson A.W., Beckerman J.L., Aime M.C. (2014). First Report of the White Pine Blister Rust Fungus, *Cronartium ribicola*, on Ribes odoratum in Indiana. Plant Dis..

[21] Miladinović B., Kostić M., Šavikin K., Đorđević B., Mihajilov-Krstev T., Živanović S., Kitić D. (2014). Chemical Profile and Antioxidative and Antimicrobial Activity of Juices and Extracts of 4 Black Currants Varieties (*Ribes nigrum* L.). J. Food Sci..

[22] Lim T.K. (2015). Edible Medicinal and Non Medicinal Plants: Volume 9, Modified Stems, Roots, Bulbs.

[23] Damascos M.A., Arribere M., Svriz M., Bran D. (2008). Fruit Mineral Contents of Six Wild Species of the North Andean Patagonia, Argentina. Biol. Trace Elem. Res..

[24] Orsavová J., Hlaváčová I., Mlček J., Snopek L., Mišurcová L. (2019). Contribution of phenolic compounds, ascorbic acid and vitamin E to antioxidant activity of currant (*Ribes* L.) and gooseberry (*Ribes uva-crispa* L.) fruits. Food Chem..

[25] Xiang J.-M., Zhong G.-Y., Jiang W., Ren G. (2021). Analysis of varieties and standards of Saxifragaceae medicinal plants used in Tibetan medicine. Zhongguo Zhong Yao Za Zhi.

[26] Kan J., Wang M., Liu Y., Liu H., Chen L., Zhang X., Huang C., Liu B.Y., Gu Z., Du J. (2020). A novel botanical formula improves eye fatigue and dry eye: A randomized, double-blind, placebo-controlled study. Am. J. Clin. Nutr..

[27] Uttra A.M., Alamgeer, Shahzad M., Shabbir A., Jahan S., Bukhari I.A., Assiri A.M. (2019). Ribes orientale: A novel therapeutic approach targeting rheumatoid arthritis with reference to pro-inflammatory cytokines, inflammatory enzymes and anti-inflammatory cytokines. J. Ethnopharmacol..

[28] Ferlemi A.-V., Lamari F. (2016). Berry Leaves: An Alternative Source of Bioactive Natural Products of Nutritional and Medicinal Value. Antioxidants.

[29] Birasuren B., Oh H.L., Kim C.R., Kim N.Y., Jeon H.L., Kim M.R. (2012). Antioxidant Activities of *Ribes diacanthum* Pall Extracts in the Northern Region of Mongolia. JFN.

[30] Kim H.J., Baek S.Y., Sok D.-E., Lee K.J., Kim Y.-J., Kim M.R. (2020). Neuroprotective Activity of Polyphenol-Rich *Ribes diacanthum* Pall Against Oxidative Stress in Glutamate-Stimulated HT-22 Cells and a Scopolamine-Induced Amnesia Animal Model. Antioxidants.

[31] Khan S.W., Khatoon S. (2007). Ethnobotanical studies on useful trees and shrubs of haramosh and bugrote valleys, in Gilgit northern areas of Pakistan. Pak. J. Bot..

[32] Sadia H., Zafar M., Ahmad M., Lubna, Khan M.P.Z., Yaseen G., Ali M.I., Sultana S., Kilic O., Şahan Z. (2020). Foliar epidermal anatomy of some selected wild edible fruits of Pakistan using light microscopy and scanning electron microscopy. Microsc. Res Tech..

[33] Lim T.K. (2012). Edible Medicinal and Non-Medicinal Plants: Volume 4, Fruits.

[34] Hassan U.H., Alamgeer, Shahzad M., Shabbir A., Jahan S., Saleem M., Bukhari I.A., Assiri A.M. (2019). Amelioration of adjuvant induced arthritis in Sprague Dawley rats through modulation of inflammatory mediators by *Ribes alpestre* Decne. J. Ethnopharmacol..

[35] Abbas Q., Khan S.W., Khatoon S., Hussain S.A., Hassan S.N., Hussain A., Qureshi R., Hussain I. (2014). Floristic biodiversıty and traditional uses of medicinal plants of haramosh valley central karakoram national Park of Gilgit district, gilgit-baltistan. J. Biodivers. Environ. Sci. (JBES).

[36] Khan B., Abdukadir A., Qureshi R., Mustafa G. (2011). Medicinal uses of plants by the inhabitants of Khunjerab National park, Gilgit, Pakistan. Pak. J. Bot..

[37] Committee on Herbal Medicinal Products (HMPC) (2009). Community Herbal Monograph on Ribes nigrum L. Folium.

[38] Sun X., Zhan Y., Li S., Liu Y., Fu Q., Quan X., Xiong J., Gang H., Zhang L., Qi H. (2023). Complete chloroplast genome assembly and phylogenetic analysis of blackcurrant (*Ribes nigrum*), red and white currant (*Ribes rubrum*), and gooseberry (*Ribes uva-crispa*) provide new insights into the phylogeny of Grossulariaceae. PeerJ.

[39] Zhang B., Yu Z., Xu Z., Zheng B. (2023). A Phylogenetic and Morphological Evolution Study of *Ribes* L. in China Using RAD-Seq. Plants.

[40] Schultheis L.M., Donoghue M.J. (2004). Molecular Phylogeny and Biogeography of Ribes (Grossulariaceae), with an Emphasis on Gooseberries (subg. Grossularia). Syst. Bot..

[41] Kłubowicz K., Sawicki J., Paukszto Ł., Ciborowski K., Maździarz M., Krawczyk K. (2024). Organellar genome evolution in *Ribes* L.: Hotspots of mutation, sequence transfer, codon usage, and phylogenetic context. Tree Genet. Genomes.

[42] Russell J.R., Bayer M., Booth C., Cardle L., Hackett C.A., Hedley P.E., Jorgensen L., Morris J.A., Brennan R.M. (2011). Identification, utilisation and mapping of novel transcriptome-based markers from blackcurrant (*Ribes nigrum*). BMC Plant Biol..

[43] Sun M., Lin Q. (2010). A revision of *Elaeagnus* L. (Elaeagnaceae) in mainland China. J. Syst. Evol..

[44] Luckow M. (1995). Species Concepts: Assumptions, Methods, and Applications. Syst. Bot..

[45] Chan K.O., Hutter C.R., Wood P.L., Grismer L.L., Brown R.M. (2020). Larger, unfiltered datasets are more effective at resolving phylogenetic conflict: Introns, exons, and UCEs resolve ambiguities in Golden-backed frogs (Anura: Ranidae; genus Hylarana). Mol. Phylogenetics Evol..

[46] Senters A.E., Soltis D.E. (2003). Phylogenetic Relationships in *Ribes* (Grossulariaceae) Inferred from ITS Sequence Data. Taxon.

[47] Lu R.-S., Yang T., Chen Y., Wang S.-Y., Cai M.-Q., Cameron K.M., Li P., Fu C.-X. (2021). Comparative plastome genomics and phylogenetic analyses of Liliaceae. Bot. J. Linn. Soc..

[48] Cui X., Liu K., Li E., Zhang Z., Dong W. (2024). Chloroplast Genomes Evolution and Phylogenetic Relationships of Caragana species. Int. J. Mol. Sci..

[49] Bohm B.A. (1993). External and vacuolar flavonoids of *Ribes viscossisimum*. Biochem. Syst. Ecol..

[50] Farooque S., Rose P.M., Benohoud M., Blackburn R.S., Rayner C.M. (2018). Enhancing the Potential Exploitation of Food Waste: Extraction, Purification, and Characterization of Renewable Specialty Chemicals from Blackcurrants (*Ribes nigrum* L.). J. Agric. Food Chem..

[51] Slimestad R., Solheim H. (2002). Anthocyanins from Black Currants (*Ribes nigrum* L.). J. Agric. Food Chem..

[52] Anttonen M.J., Karjalainen R.O. (2006). High-Performance Liquid Chromatography Analysis of Black Currant (*Ribes nigrum* L.) Fruit Phenolics Grown either Conventionally or Organically. J. Agric. Food Chem..

[53] Määttä K.R., Kamal-Eldin A., Törrönen A.R. (2003). High-Performance Liquid Chromatography (HPLC) Analysis of Phenolic Compounds in Berries with Diode Array and Electrospray Ionization Mass Spectrometric (MS) Detection: Ribes Species. J. Agric. Food Chem..

[54] Sandell M., Laaksonen O., Järvinen R., Rostiala N., Pohjanheimo T., Tiitinen K., Kallio H. (2009). Orosensory Profiles and Chemical Composition of Black Currant (*Ribes nigrum*) Juice and Fractions of Press Residue. J. Agric. Food Chem..

[55] Nanashima N., Horie K., Yamanouchi K., Tomisawa T., Kitajima M., Oey I., Maeda H. (2020). Blackcurrant (*Ribes nigrum*) Extract Prevents Dyslipidemia and Hepatic Steatosis in Ovariectomized Rats. Nutrients.

[56] Haasbach E., Hartmayer C., Hettler A., Sarnecka A., Wulle U., Ehrhardt C., Ludwig S., Planz O. (2014). Antiviral activity of Ladania067, an extract from wild black currant leaves against influenza A virus in vitro and in vivo. Front. Microbiol..

[57] Laczkó-Zöld E., Komlósi A., Ülkei T., Fogarasi E., Croitoru M., Fülöp I., Domokos E., Ştefănescu R., Varga E. (2018). Extractability of polyphenols from black currant, red currant and gooseberry and their antioxidant activity. Acta Biol. Hung..

[58] Li L., Hwang E., Ngo H.T.T., Seo S.A., Lin P., Gao W., Liu Y., Yi T. (2018). *Ribes nigrum* L. Prevents UVB-mediated Photoaging in Human Dermal Fibroblasts: Potential Antioxidant and Antiinflammatory Activity. Photochem. Photobiol..

[59] Razgonova M.P., Nawaz M.A., Sabitov A.S., Golokhvast K.S. (2024). Genus Ribes: *Ribes aureum*, *Ribes pauciflorum*, *Ribes triste*, and *Ribes dikuscha*—Comparative Mass Spectrometric Study of Polyphenolic Composition and Other Bioactive Constituents. Int. J. Mol. Sci..

[60] Zhao Y., Lu H., Wang Q., Liu H., Shen H., Xu W., Ge J., He D. (2021). Front Cover: Rapid qualitative profiling and quantitative analysis of phenolics in *Ribes meyeri* leaves and their antioxidant and antidiabetic activities by HPLC-QTOF-MS/MS and UHPLC-MS/MS. J. Sep. Sci..

[61] Li Y., Zhou B., Zhang W., Yang G., Zhang C., Cao Z. (2018). Chemical constituents from aerial parts of *Ribes mandshuricum*. Chin. Tradit. Herb. Drugs.

[62] Zhang L., Wang Q., Zhao Y., Ge J., He D. (2023). Phenolic Profiles, Antioxidant, and Hypoglycemic Activities of *Ribes meyeri* Fruits. Foods.

[63] Liu C., Lei Y., Liu Y., Guo J., Chen X., Tang Y., Dang J., Wu M. (2023). An Integrated Strategy for Investigating Antioxidants from *Ribes himalense* Royle ex Decne and Their Potential Target Proteins. Antioxidants.

[64] Knox Y.M., Suzutani T., Yosida I., Azuma M. (2003). Anti-influenza virus activity of crude extract of *Ribes nigrum* L.. Phytother. Res..

[65] Staszowska-Karkut M., Materska M. (2020). Phenolic Composition, Mineral Content, and Beneficial Bioactivities of Leaf Extracts from Black Currant (*Ribes nigrum* L.), Raspberry (*Rubus idaeus*), and Aronia (*Aronia melanocarpa*). Nutrients.

[66] Yang H., Bai J., Ma C., Wang L., Li X., Zhang Y., Xu Y., Yang Y. (2020). Degradation models, structure, rheological properties and protective effects on erythrocyte hemolysis of the polysaccharides from *Ribes nigrum* L.. Int. J. Biol. Macromol..

[67] Zhou B., Zhang C., Zou X., Xu J., Li Y., Li X., Chai C., Cao Z. (2016). Chemical constituents from the aerial Parts of *Ribes diacanthum* Pall. Chin. Pharmaceut. J..

[68] Dvaranauskaite A., Venskutonis P.R., Raynaud C., Talou T., Viškelis P., Dambrauskiene E. (2008). Characterization of Steam Volatiles in the Essential Oil of Black Currant Buds and the Antioxidant Properties of Different Bud Extracts. J. Agric. Food Chem..

[69] Ðorđević B.S., Pljevljakušić D.S., Šavikin K.P., Stević T.R., Bigović D.J. (2014). Essential Oil from Blackcurrant Buds as Chemotaxonomy Marker and Antimicrobial Agent. Chem. Biodivers..

[70] Oprea E., Farcasanu I.C., Radulescu V., Balotescu C., Bucur M., Lazar V., Mladin P. (2008). Chemical and biological studies of *Ribes nigrum* L. buds essential oil. Biofactors.

[71] Zhao M., Bai J., Bu X., Yin Y., Wang L., Yang Y., Xu Y. (2021). Characterization of selenized polysaccharides from *Ribes nigrum* L. and its inhibitory effects on α-amylase and α-glucosidase. Carbohydr. Polym..

[72] Yang Y., Zou J., Li M., Yun Y., Li J., Bai J. (2024). Extraction and characterization of polysaccharides from blackcurrant fruits and its inhibitory effects on acetylcholinesterase. Int. J. Biol. Macromol..

[73] Huang X., Zheng S., Guo Y., Yu B., Zhao M., Guo P., Bai J., Yang Y. (2025). Structure characterization of polysaccharide isolated from *Ribes nigrum* L. and it’s bioactivity against gout. Int. J. Biol. Macromol..

[74] Burgos-Edwards A., Theoduloz C., Miño S., Ghosh D., Shulaev V., Ramírez C., Sánchez-Jardón L., Rozzi R., Schmeda-Hirschmann G. (2024). Phenolic composition and bioactivity of *Ribes magellanicum* fruits from southern Patagonia. Heliyon.

[75] Lappi J., Raninen K., Väkeväinen K., Kårlund A., Törrönen R., Kolehmainen M. (2021). Blackcurrant (*Ribes nigrum*) lowers sugar-induced postprandial glycaemia independently and in a product with fermented quinoa: A randomised crossover trial. Br. J. Nutr..

[76] Dashti S., Hadjzadeh M.A., Ghorbani A., Mohebbi M., Gholamnezhad Z. (2022). The antihyperglycemic and hypolipidemic effects of *Ribes khorassanicum* hydro-ethanolic extract co-administration in type 2 diabetic patients: A randomized double blind placebo controlled trial. Avicenna J. Phytomed..

[77] Da Costa P., Schetinger M.R.C., Baldissarelli J., Stefanello N., Lopes T.F., Reichert K.P., Assmann C.E., Bottari N.B., Miron V.V., Vargas F.F.A. (2024). Blackcurrant (*Ribes nigrum* L.) improves cholinergic signaling and protects against chronic Scopolamine-induced memory impairment in mice. J. Psychopharmacol..

[78] Shimada M., Maeda H., Nanashima N., Yamada K., Nakajima A. (2022). Anthocyanin-rich blackcurrant extract improves long-term memory impairment and emotional abnormality in senescence-accelerated mice. J. Food Biochem..

[79] Lomiwes D., Günther C.S., Bloor S.J., Trower T.M., Ngametua N., Kanon A.P., Jensen D.A., Lo K., Sawyer G., Walker E.G. (2024). Identification of Sarmentosin as a Key Bioactive from Blackcurrants (*Ribes nigrum*) for Inhibiting Platelet Monoamine Oxidase in Humans. J. Agric. Food Chem..

[80] Jiang Z., Yang X., Han Y., Li J., Hu C., Liu C., Xiao W. (2022). Sarmentosin promotes USP17 and regulates Nrf2-mediated mitophagy and cellular oxidative stress to alleviate APAP-induced acute liver failure. Phytomedicine.

[81] Lyashenko S., López-Ruiz R., García-Cervantes A.M., Rodríguez-García I., Yunusova S., Guil-Guerrero J.L. (2024). Phenolic Profiles and Antitumor Activity Against Colorectal Cancer Cells of Seeds from Selected Ribes Taxa. Appl. Sci..

[82] Nosal B.M., Thornton S.N., Mofrad M.D., Sakaki J.R., Mahoney K.J., Macdonald Z., Daddi L., Tran T.D.B., Weinstock G., Zhou Y. (2024). Blackcurrants shape gut microbiota profile and reduce risk of postmenopausal osteoporosis via the gut-bone axis: Evidence from a pilot randomized controlled trial. J. Nutr. Biochem..

[83] Amini A.M., Zhou R., Austermann K., Králová D., Serra G., Ibrahim I.S., Corona G., Bergillos-Meca T., Aboufarrag H., A Kroon P. (2025). Acute Effects of an Anthocyanin-Rich Blackcurrant Beverage on Markers of Cardiovascular Disease Risk in Healthy Adults: A Randomized, Double-Blind, Placebo-Controlled, Crossover Trial. J. Nutr..

[84] Lengsfeld C., Deters A., Faller G., Hensel A. (2004). High Molecular Weight Polysaccharides from Black Currant Seeds Inhibit Adhesion of Helicobacter pylori to Human Gastric Mucosa. Planta Med..

[85] Krisch J., Ördögh L., Galgóczy L., Papp T., Vágvölgyi C. (2009). Anticandidal effect of berry juices and extracts from *Ribes* species. Open Life Sci..

[86] Bendokas V., Šarkinas A., Jasinauskienë D., Anisimovienë N., Morkûnaitë-Haimi Š., Stanys V., Šikšnianas T. (2018). Antimicrobial activity of berries extracts of four Ribes species, their phenolic content and anthocyanin composition. Folia Hortic..

[87] Hovhannisyan Z., Timotina M., Manoyan J., Gabrielyan L., Petrosyan M., Kusznierewicz B., Bartoszek A., Jacob C., Ginovyan M., Trchounian K. (2022). *Ribes nigrum* L. Extract-Mediated Green Synthesis and Antibacterial Action Mechanisms of Silver Nanoparticles. Antibiotics.

[88] Sermukhamedova O., Wojtanowski K.K., Widelski J., Korona-Głowniak I., Elansary H.O., Sakipova Z., Malm A., Głowniak K., Skalicka-Woźniak K. (2017). Metabolic Profile of and Antimicrobial Activity in the Aerial Part of *Leonurus turkestanicus* V.I. Krecz. et Kuprian. from Kazakhstan. J. AOAC Int..

[89] Sabitov A., Gaweł-Bęben K., Sakipova Z., Strzępek-Gomółka M., Hoian U., Satbayeva E., Głowniak K., Ludwiczuk A. (2021). *Rosa platyacantha* Schrenk from Kazakhstan—Natural Source of Bioactive Compounds with Cosmetic Significance. Molecules.

[90] Gao J., Wu Y., He D., Zhu X., Li H., Liu H., Liu H. (2020). Anti-aging effects of *Ribes meyeri* anthocyanins on neural stem cells and aging mice. Aging.

[91] Shaposhnik E.I., Deineka L.A., Sorokopudov V.N., Deineka V.I., Burmenko Y.V., Kartushinsky V.V., Tregubov A.V. (2011). Biologically active substances of *Ribes* l. fruits. Reg. Geosystems.

[92] Gülmez G., Şen A., Şekerler T., Algül F.K., Çilingir-Kaya Ö.T., Şener A. (2022). The antioxidant, anti-inflammatory, and antiplatelet effects of *Ribes rubrum* L. fruit extract in the diabetic rats. J. Food Biochem..

[93] Minasyan A., Pires V., Gondcaille C., Ginovyan M., Mróz M., Savary S., Cherkaoui-Malki M., Kusznierewicz B., Bartoszek A., Andreoletti P. (2025). Ribes nigrum leaf extract downregulates pro-inflammatory gene expression and regulates redox balance in microglial cells. BMC Complement Med. Ther..

[94] Magnavacca A., Piazza S., Cammisa A., Fumagalli M., Martinelli G., Giavarini F., Sangiovanni E., Dell’Agli M. (2021). Ribes Nigrum Leaf Extract Preferentially Inhibits IFN-γ-Mediated Inflammation in HaCaT Keratinocytes. Molecules.

[95] Garbacki N., Tits M., Angenot L., Damas J. (2004). Inhibitory Effects of Proanthocyanidins from *Ribes nigrum* Leaves on Carrageenin Acute Inflammatory Reactions Induced in Rats. BMC Pharmacol..

[96] Karlsen A., Retterstøl L., Laake P., Paur I., Kjølsrud-Bøhn S., Sandvik L., Blomhoff R. (2007). Anthocyanins Inhibit Nuclear Factor-κB Activation in Monocytes and Reduce Plasma Concentrations of Pro-Inflammatory Mediators in Healthy Adults, 3. J. Nutr..

[97] Benn T., Kim B., Park Y.-K., Yang Y., Pham T.X., Ku C.S., Farruggia C., Harness E., Smyth J.A., Lee J.-Y. (2015). Polyphenol-rich blackcurrant extract exerts hypocholesterolaemic and hypoglycaemic effects in mice fed a diet containing high fat and cholesterol. Br. J. Nutr..

[98] Benn T., Kim B., Park Y.-K., Wegner C.J., Harness E., Nam T.-G., Kim D.-O., Lee J.S., Lee J.-Y. (2014). Polyphenol-rich blackcurrant extract prevents inflammation in diet-induced obese mice. J. Nutr. Biochem..

[99] Lee Y., Pham T.X., Bae M., Hu S., O’Neill E., Chun O.K., Han M.J., Koo S.I., Park Y.-K., Lee J.-Y. (2019). Blackcurrant (*Ribes nigrum*) Prevents Obesity-Induced Nonalcoholic Steatohepatitis in Mice. Obesity.

[100] Lee Y., Lee J.Y. (2019). Blackcurrant (*Ribes nigrum*) Extract Exerts an Anti-Inflammatory Action by Modulating Macrophage Phenotypes. Nutrients.

